# Supplementary material for: XopA: a novel type III secretion system effector in Xenorhabdus that modulates host cell responses through apoptosis, autophagy, and immune evasion
Source: Microbiol Spectr. 2026 Feb 24;14(4):e03871-25. doi: 10.1128/spectrum.03871-25 (PMC13055249; doi:10.1128/spectrum.03871-25)

**Supplementary information**

**XopA: A Novel Type III Secretion System Effector in *Xenorhabdus* that Modulates Host Cell Responses through Apoptosis, Autophagy, and Immune Evasion**

Xiyin Huang^1^, Xingya Dong^1^, Chen Li^2^, Jiajie Xie^2^, Yunjun Sun^2^, Yibo Hu^2^, Liqiu Xia^2^, Qiang Tu^1^*, Youming Zhang^1,3^, Shengbiao Hu^2^*

1: *Institute of Synthetic Biology Industry (College of Synthetic Biology Industry), Hunan University of Arts and Science, Changde 415000, China*;

2: *State Key Laboratory of Developmental Biology of Freshwater Fish, Hunan Provincial Key Laboratory of Microbial Molecular Biology, College of Life Science, Hunan Normal University, Changsha, China*;

3: *Helmholtz International Lab for Anti-infectives, Shandong University–Helmholtz Institute of Biotechnology, State Key Laboratory of Microbial Technology, Shandong University, Qingdao, China*

*Correspondence

College of Life Science, Hunan Normal University, No.36 Lushan Street, Changsha 410081, China.

Emails: [shengbiaohu@hunnu.edu.cn](mailto:shengbiaohu@hunnu.edu.cn)

**Table S1 Primers used in this study**(The primer sequences were synthesized by Shanghai Sangon Biotechnology Co., Ltd.).

| **Primers** | **Primer sequences(5'-3')** | **Usage** |
| --- | --- | --- |
| **XopA-F** | **AGATCTCGAGCTCTACTTTGATATTAAATATATC** | **Construction of XopA eukaryotic expression vector.** |
| **XopA-R** | **GATCACTAGTCAAAAAACCCCTCAAGACCCGTTTAGAGGCCCCAAGGGGTTATGCTAGTTACTTGTACAGCTCGTCCATGC** | **Construction of XopA eukaryotic expression vector.** |
| **Q_152_A-F** | **GAGTAATCAAGCAAATAGTATTGGTGAATGTATTATCTATAGCTT** | **Construction of XopA amino acid site mutation vector.** |
| **Q_152_A-R** | **CTATTTGCTTGATTACTCGATAAAATACGTAAAGAAA** | **Construction of XopA amino acid site mutation vector.** |
| **S_154_A-F** | **AACAAAATGCTATTGGTGAATGTATTATCTATAGCTTATCTTT** | **Construction of XopA amino acid site mutation vector.** |
| **S_154_A-R** | **CACCAATAGCATTTTGTTGATTACTCGATAAAATACGTAA** | **Construction of XopA amino acid site mutation vector.** |
| **C_158_A-F** | **TTGGTGAAGCTATTATCTATAGCTTATCTTTAGTAAAGAAGATGC** | **Construction of XopA amino acid site mutation vector.** |
| **C_158_A-R** | **AGATAATAGCTTCACCAATACTATTTTGTTGATTACTCG** | **Construction of XopA amino acid site mutation vector.** |
| **L_163_A-F** | **TCTATAGCGCATCTTTAGTAAAGAAGATGCATAAAGAAAGT** | **Construction of XopA amino acid site mutation vector.** |
| **L_163_A-R** | **CTAAAGATGCGCTATAGATAATACATTCACCAATACTATTTTG** | **Construction of XopA amino acid site mutation vector.** |
| **N_183_A-F** | **CATCAAAAAGCCCTTAATAATGAACTAAATATTACGCATAAA** | **Construction of XopA amino acid site mutation vector.** |
| **N_183_A-R** | **ATTAAGGGCTTTTTGATGTAAAAGATAGAGCTTTTCA** | **Construction of XopA amino acid site mutation vector.** |
| **ATG5-F** | **TCAGAAGGTTATGAGACAAG** | **RT-PCR amplification of *atg5*.** |
| **ATG5-R** | **TAGATGGACAGTGCAGAAGG** | **RT-PCR amplification of *atg5*.** |
| **Caspase-3-F** | **TGGATTATCCTGAGATGGGT** | **RT-PCR amplification of *caspase-3*.** |
| **Caspase-3-R** | **ACATCACGCATCAATTCCAC** | **RT-PCR amplification of *caspase-3*.** |
| **β-Actin-F** | **ATCTGGCACCACACCTTCTA** | **RT-PCR amplification of *β-actin*.** |
| **β-Actin-R** | **AGAGGCGTACAGGGATAGCA** | **RT-PCR amplification of *β-actin*.** |


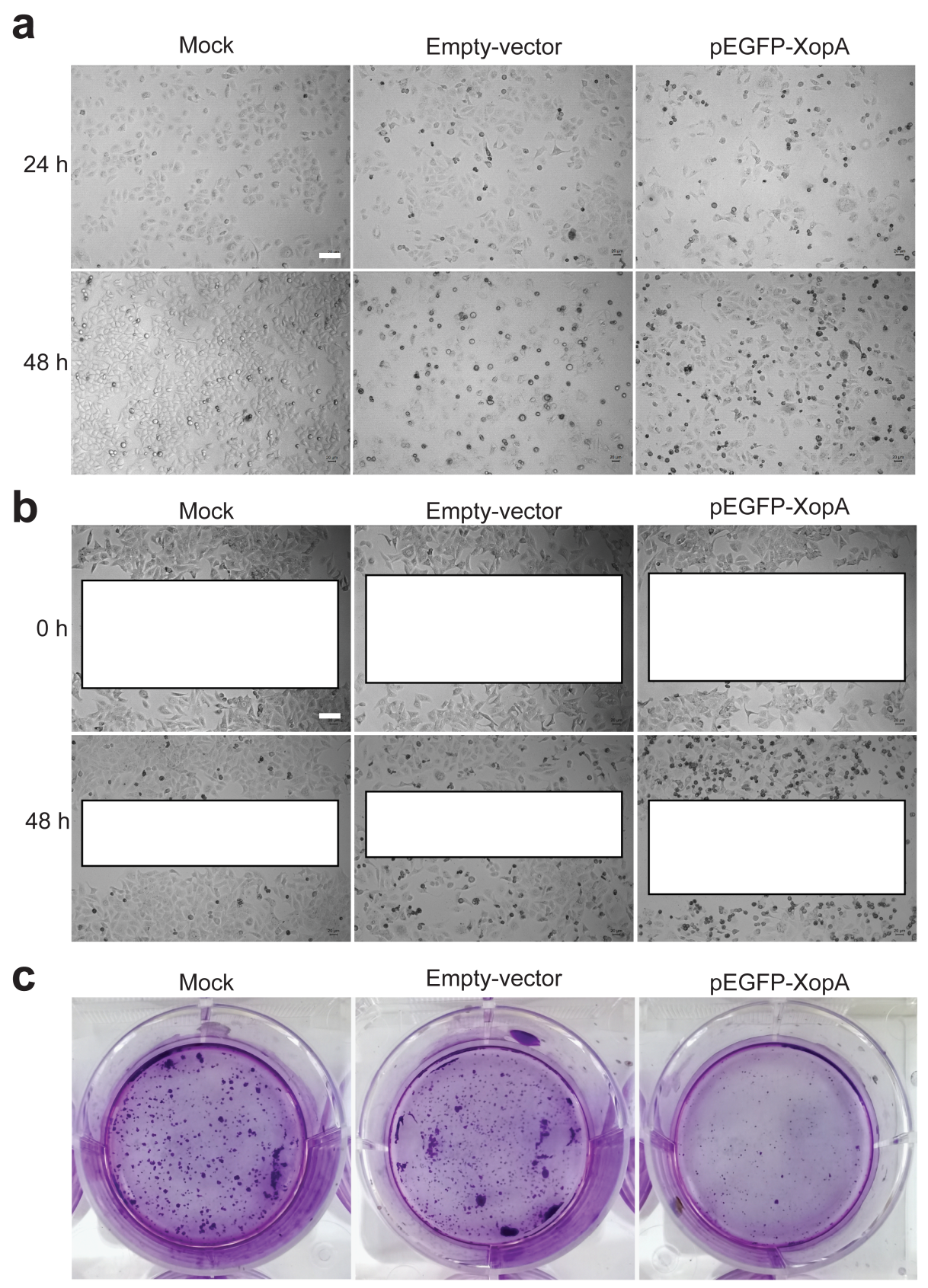
**Fig.S1 XopA induces cytotoxicity and inhibits cell proliferation.**

**(a)** The cell morphology was observed using an inverted microscope. HeLa cells were transfected for 24 h and 48 h, and images were captured using the inverted microscope. Scale bars, 20 μm. **(b)** Scratch repair experiment. The cells in each group were left with equal width scratches, and the width of the scratches was compared after 48 h of culture. Scale bars, 20 μm. Experiments were repeated twice with similar results. **(c)** The number and size of cell clones formed after 14 days of plasmid transfection into HeLa cells.


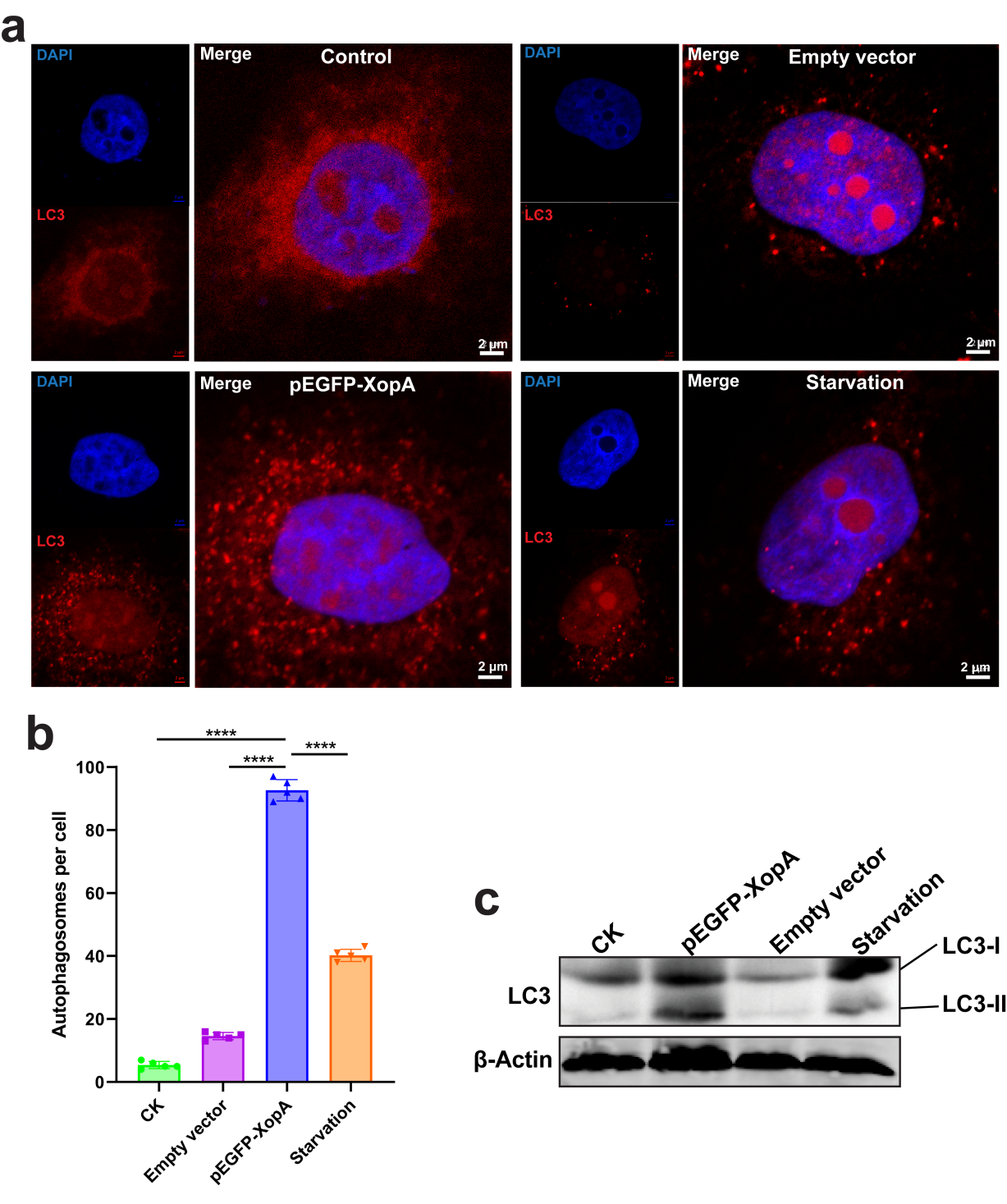
**Fig.S2 XopA induced autophagy in HEK-293T cells.**

During the experiment, HEK-293T cells were treated under the same conditions as the HeLa cells in **Fig.4**. **(a)** The occurrence of autophagy was detected through immunofluorescence staining. After transfecting HEK-293T cells for 24 h, LC3 was labeled as red and the nucleus was labeled as blue using immunofluorescence labeling. The bright red spots observed in the confocal microscope image represent autophagic spots. Scale bars, 1 μm. **(b)** Quantification of autophagic spots. The number of LC3 bright spots in each cell in **(a)** was quantified. The data presented in the chart represents the average value of 5 result groups, with each group consisting of data from 5 fields of view. **(c)** The occurrence of autophagy was detected through immunoblotting. The lysates of HEK-293T cells, 24 h after transfection, were analyzed using immunoblotting with LC3 antibody. Experiments were repeated 3 times with similar results. The results were shown as the mean ± S.D. ****P< 0.0001.


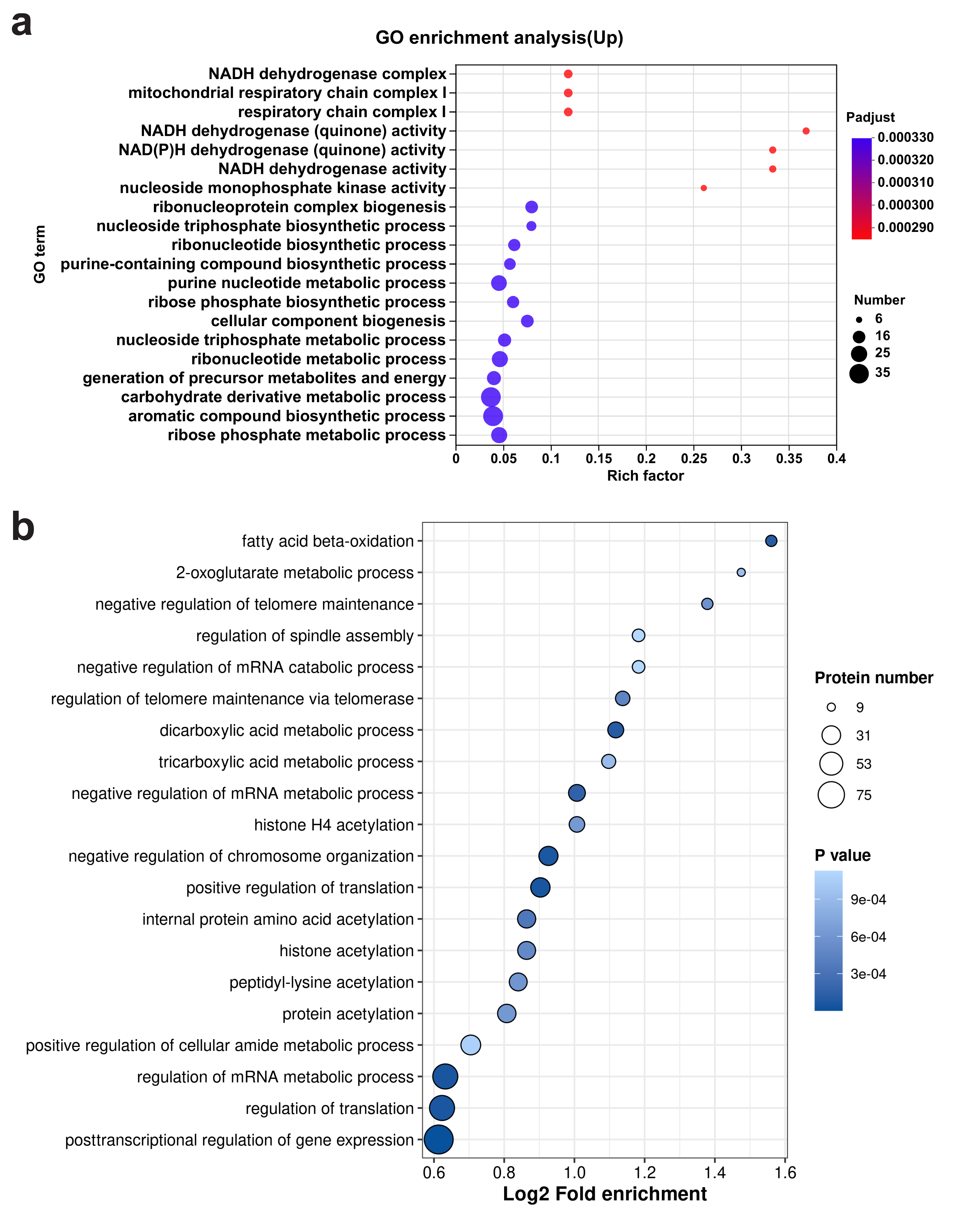
**Fig.S3 XopA promotes cellular metabolism and processes.**

GO enrichment analysis of up-regulated genes was performed based on transcriptional level of RAW264.7 cells and acetylation modification level of HeLa cells after XopA expression. **(a)** GO enrichment analysis of up-regulated gene in RAW264.7 cells after XopA expression for 18 h. **(b)** GO enrichment analysis of up-regulated genes in HeLa cells after XopA expression for 18 h.

**Fig.S4 XopA promotes cell metabolism to provide sufficient acetylation reaction substrates.**

KEGG enrichment analysis of upregulated genes was performed based on transcription levels of RAW264.7 cells and acetylation modification levels of HeLa cells after XopA expression. **(a)** KEGG enrichment analysis of up-regulated gene in RAW264.7 cells after XopA expression for 18 h. **(b)** KEGG enrichment analysis of up-regulated genes in HeLa cells after XopA expression for 18 h.
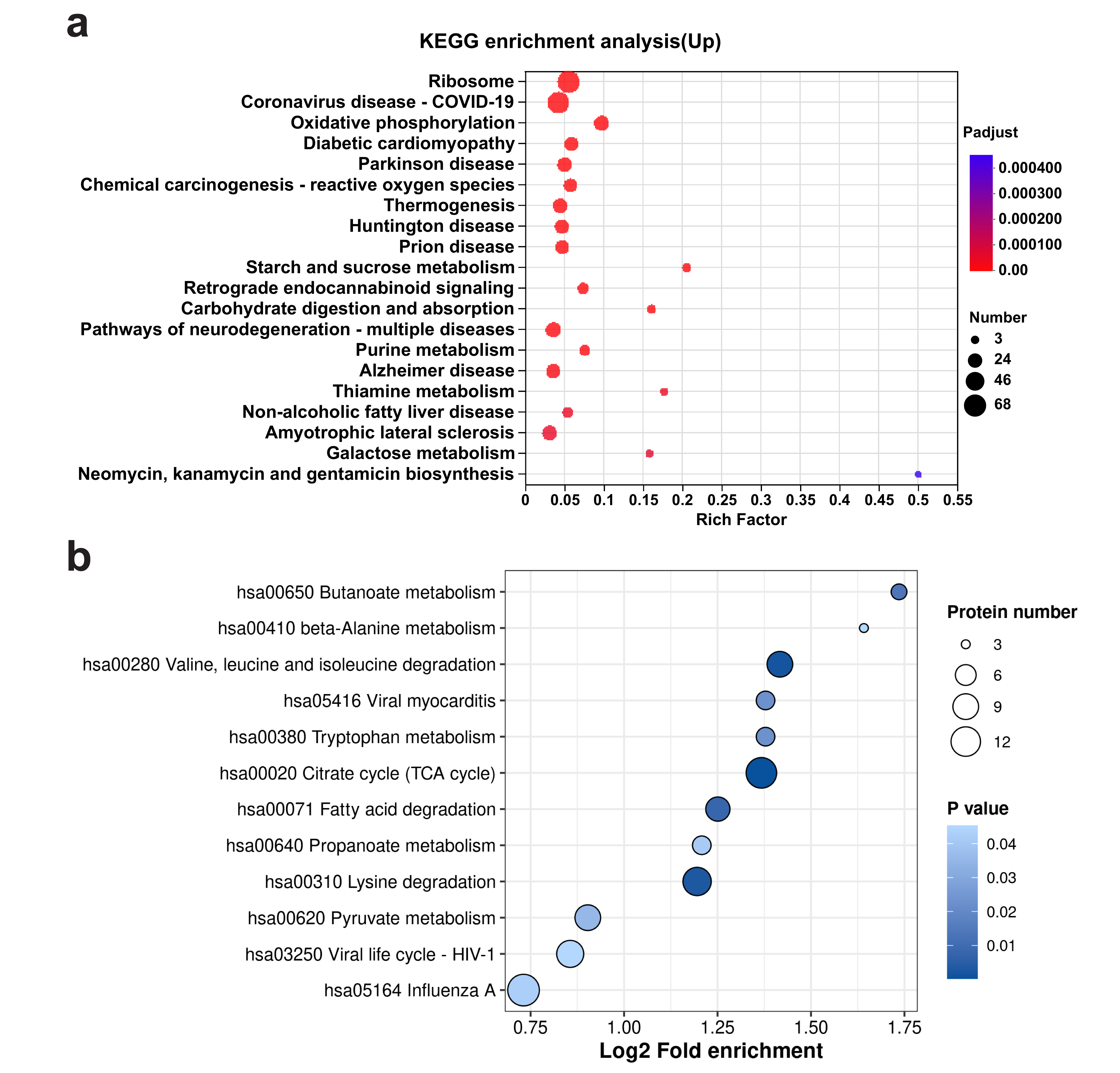

Supplement: Table S1 and Figures S1 to S4 — Table S1: Primers used in this study. Figure S1: XopA induces cytotoxicity and inhibits cell proliferation. Figure S2: XopA induced autophagy in HEK-293T cells. Figure S3: XopA promotes cellular metabolism and processes. Figure S4: XopA promotes cell metabolism to provide sufficient acetylation reaction substrates. [file spectrum.03871-25-s0001.docx]
